# Diagnosis of a Stress Fracture Of a Metatarsal Bone with Point of Care Ultrasound (POCUS)

**DOI:** 10.24908/pocus.v9i2.17393

**Published:** 2024-11-15

**Authors:** Alfred Doblinger

**Affiliations:** 1 Institute of General Medicine, Medical University of Innsbruck Innsbruck AUT

**Keywords:** Stress Fracture, March Fracture, Musculoskeletal Ultrasound

## Abstract

The early diagnosis of stress fractures is a challenge in daily primary care practice. Point of care ultrasound (POCUS) can be helpful in the diagnosis of early signs of an incipient stress fracture. A 52-year-old woman presented with a history of chronic overuse in the left forefoot. A sonographic examination in the area of the reported pain point on the third metatarsal (consistent with a marching fracture) showed a clear accumulation of fluid, corresponding to subperiosteal hematoma. After only two weeks of resting the forefoot, a repeat examination using POCUS showed clear callus formation. This confirmed the suspected diagnosis of an early stage stress fracture. This case shows a sensible and easy-to-learn way of using POCUS in general practice. POCUS in combination with clinical examination and anamnesis is a cost-effective and timely diagnostic option without radiation exposure.

## Background

Diagnosing stress fractures at an early stage is a major challenge in everyday general practice. Patients present with unclear pain around a bone, often in the metatarsal region. Conventional X-rays typically only reveal the fracture after the bone has broken. A clear diagnosis before the bone breaks is only possible with an MRI, which is the gold standard 1. However, this is only immediately available to a limited extent in everyday clinical practice. An alternative option is the use of point of care ultrasound (POCUS). The diagnosis of bone fractures by ultrasound has a sensitivity described overall as 42.11 - 100% and a specificity as 65.0 - 100%, and the most accurate results in the lower extremity 2. POCUS achieves very good results (sensitivity 90.2%, specificity 96.1%), particularly in the diagnosis of traumatic long bone fractures 3.

On ultrasound, the fracture line of a bone fracture typically appears as an interruption of the corticalis. It may also occasionally appear as a dislocation of the fracture 4, whereby a hematoma forms in the area of the fracture gap in the tissue adjacent to the bone or subperiosteally. In the present case, the suspected diagnosis of a fatigue fracture in the region of a metatarsal bone could only be presumed in the POCUS based on this typical fluid accumulation, even before a complete dislocated fracture could develop.

## Case presentation

A 52-year-old woman presented to the general practitioner's office with pain in the left forefoot. The pain had been present for several weeks but had increased significantly in the last few days. Standing on her toes on request was very painful, but possible. As a sales clerk, she stands or walks for an average of eight hours a day without being able to sit down. Her medical history included a right-side metatarsal stress fracture five years ago, but otherwise no relevant diagnoses or premedication.

The clinical examination showed a clear, easily localized pressure pain in the area of the third metatarsal bone on the left without relevant swelling or redness. In summary, an early stage stress fracture of the third metatarsal bone (march fracture) due to overloading was suspected 5. A relevant pathology was not to be expected in the early phase of a fatigue fracture without a clear fracture gap and without dislocation in a convention nal X-ray examination. An MRI as the examination of choice was not available within a reasonable time horizon.

**Figure 1:**
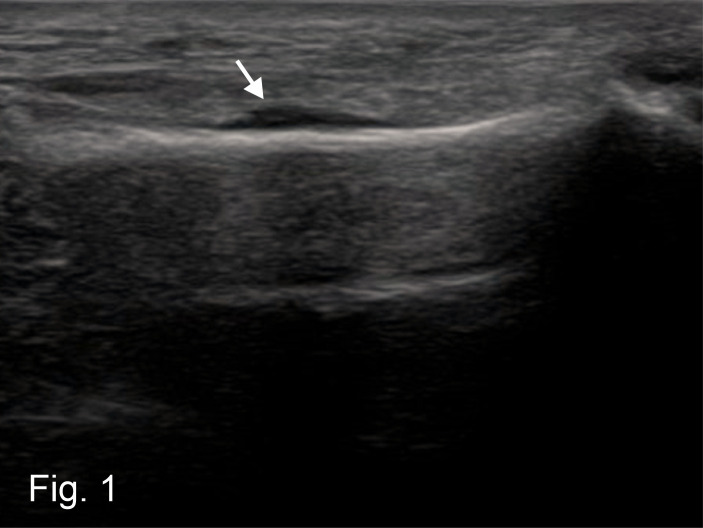
Hypoechogenic accumulation of fluid directly attached to the bone (white arrow), Hematoma between bone compacta and periosteum.

A POCUS examination performed in the area of the maximum pain point (linear probe, 12 MHZ, Alpinion E-Cube 9 Diamond), showed neither a fracture gap nor a dislocation in the corticalis area of the affected metatarsal bone. However, it did show a clearly visible accumulation of fluid directly attached to the bone (Figure 1). This was interpreted as a subperiosteal hematoma corresponding to an early stage of a stress fracture, as first described sonographically by Banal et al. in 2008 6. The patient was prescribed consistent physical rest and complete relief of the foot.

**Figure 2:**
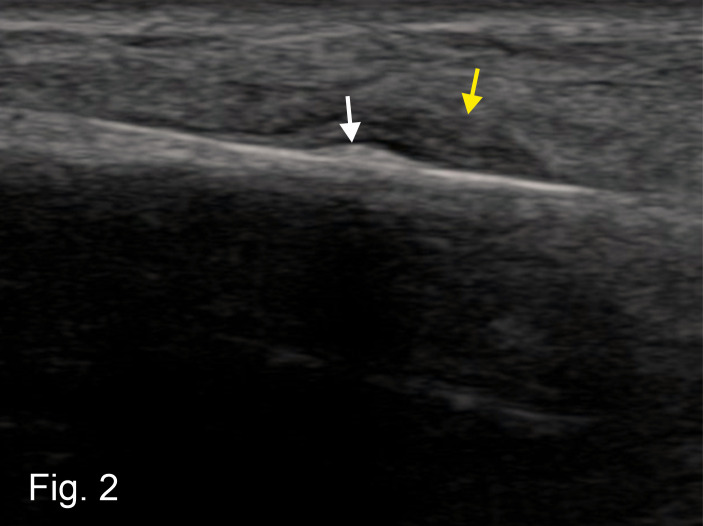
Hyperechogenic incipient callus formation (white arrow) , surrounded by a larger hypoechogenic zone matching a haematoma in organization (yellow arrow).

At a follow-up examination 14 days later, the patient was able to walk again largely without pain. In the follow-up POCUS examination, the area of the original fluid accumulation (hematoma) showed a zone matching the echogenicity of an incipient callus formation, surrounded by a larger hypoechogenic zone (Figure 2). This confirmed the diagnosis of an early stage stress fracture 7. After a further two weeks of physical rest with partial weight bearing of the forefoot, the patient was free of symptoms. After a total of four weeks, the patient was able to return to work.

## Discussion and conclusion

POCUS is a widely available, time-saving examination option in everyday general practice, with musculoskeletal ultrasound being the second most common application of POCUS 8. As early as 2008, a study showed that 30% of all MRI-based diagnoses could also be made with ultrasound 9. Since then, the quality of images on ultrasound devices have made enormous progress. In combination with the corresponding medical history and clinical presentation, the POCUS finding of an accumulation of fluid close to the bone in the form of a subperiosteal hematoma supported the clinical suspicion of an incipient stress fracture.

Recent studies have shown that in many cases of stress fractures, increased vascularization can be found using Doppler ultrasound. Immediately performing a Doppler examination when a stress fracture is clinically suspected can provide additional support for the diagnosis of an incipient stress fracture of the bone even before an actual complete fracture of the bone occurs 10, 11.

POCUS can also be used to monitor and evaluate the healing process up to the onset of callus formation with minimal time expenditure and without radiation exposure. 

## Disclosure

Consent for publication has been obtained from the patient.

## Competing interest: 

The author declares no conflict of interests.
